# Block catiomer with flexible cationic segment enhances complexation with siRNA and the delivery performance in vitro

**DOI:** 10.1080/14686996.2021.1976055

**Published:** 2021-10-13

**Authors:** Wenqian Yang, Takuya Miyazaki, Pengwen Chen, Taehun Hong, Mitsuru Naito, Yuji Miyahara, Akira Matsumoto, Kazunori Kataoka, Kanjiro Miyata, Horacio Cabral

**Affiliations:** a Department of Bioengineering, Graduate School of Engineering, The University of Tokyo, Tokyo, Japan; b Innovation Center of NanoMedicine, Kawasaki Institute of Industrial Promotion, Kawasaki, Japan; c Kanagawa Institute of Industrial Science and Technology, Kanagawa, Japan; d Institute of Biomaterials and Bioengineering, Tokyo Medical and Dental University, Tokyo, Japan; e Center for Disease Biology and Integrative Medicine, Graduate School of Medicine, The University of Tokyo, Tokyo, Japan; f Department of Materials Engineering, Graduate School of Engineering, The University of Tokyo, Tokyo, Japan

**Keywords:** Small interfering RNAs, unit polyion complexes, polyion complex vesicles, polymer flexibility, molecular dynamic simulation, 30 Bio-inspired and biomedical materials, 100 Materials, 101 Self-assembly / Self-organized materials

## Abstract

RNA interference (RNAi) by small interfering RNAs (siRNAs) is a promising therapeutic approach. Because siRNA has limited intracellular access and is rapidly cleared *in vivo*, the success of RNAi depends on efficient delivery technologies. Particularly, polyion complexation between block catiomers and siRNA is a versatile approach for constructing effective carriers, such as unit polyion complexes (uPIC), core-shell polyion complex (PIC) micelles and vesicular siRNAsomes, by engineering the structure of block catiomers. In this regard, the flexibility of block catiomers could be an important parameter in the formation of PIC nanostructures with siRNA, though its effect remains unknown. Here, we studied the influence of block catiomer flexibility on the assembly of PIC structures with siRNA using a complementary polymeric system, *i.e*. poly(ethylene glycol)-poly(L-lysine) (PEG-PLL) and PEG-poly(glycidylbutylamine) (PEG-PGBA), which has a relatively more flexible polycation segment than PEG-PLL. Mixing PEG-PGBA with siRNA at molar ratios of primary amines in polymer to phosphates in the siRNA (N/P ratios) higher than 1.5 promoted the multimolecular association of uPICs, whereas PEG-PLL formed uPIC at all N/P ratios higher than 1. Moreover, uPICs from PEG-PGBA were more stable against counter polyanion exchange than uPICs from PEG-PLL, probably due to a favorable complexation process, as suggested by computational studies of siRNA/block catiomer binding. In *in vitro* experiments, PEG-PGBA uPICs promoted effective intracellular delivery of siRNA and efficient gene knockdown. Our results indicate the significance of polycation flexibility on assembling PIC structures with siRNA, and its potential for developing innovative delivery systems.

## Graphical abstract

**Figure:**
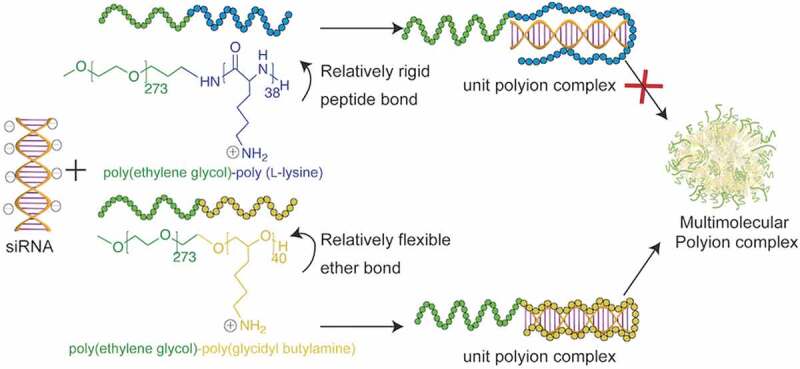

## 1. Introduction

RNA interference (RNAi) is a promising biological process with potential for developing therapies with unprecedented efficacy [1,2]. Small interfering RNAs (siRNAs), i.e. short double-stranded RNA molecules, can exert RNAi by entering the cytoplasm of cells and degrading messenger RNA (mRNA) in a sequence-specific way after being loaded in the RNA-induced silencing complex (RISC) [3,4]. However, as siRNA is a negatively charged macromolecule, its access into the cytosol is limited [5]. Moreover, siRNA is rapidly degraded and cleared in vivo [5,6]. Thus, siRNA needs to be chemically modified and/or encapsulated into nanocarriers for effective RNAi [7,8]. In this regard, polyion complex (PIC) formation between siRNA and catiomers, i.e. block copolymers having a neutral block and a positively charged segment, has been applied for self-assembling a variety of nanocarriers with auspicious characteristics, including effective siRNA loading, controlled size and nanostructure, improved intracellular delivery, and enhanced pharmacokinetics and tissue distribution [9–11]. Engineering the structure of the catiomers allows controlling the features of the PIC-based nanocarriers in a straightforward manner for promoting delivery efficiency.

**Figure 1.**
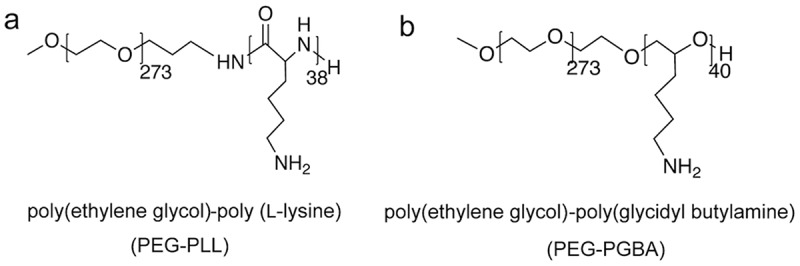
Structure of block catiomers bearing polycation segments with different flexibility. Poly(ethylene glycol)-poly(L-lysine) (PEG-PLL) presents the relatively rigid amide backbone, and poly(ethylene glycol)-poly(glycidyl butylamine) (PEG-PGBA) has a polyether backbone, which presents C-O-C bonds with low rotational barrier. These polymers have matching PEG segments and comparable polycation length

**Table 1.** Main parameters of the molecular simulation systems

System	Number of water molecules	Number of NA^+^/Cl^–^	Box volume (Å^3^)
PLL/siRNA	9845	65/75	412,000
PGBA/siRNA	10145	65/75	413,150
PEG/siRNA	12014	65/65	418,479
PEG-PLL/siRNA	17559	130/140	605,800
PEG-PGBA/siRNA	17302	130/140	595,017

The structural control of the block catiomers is decisive for modulating their complexation with siRNA and the secondary association of siRNA-catiomer complexes into multimolecular assemblies [12–18]. Thus, the control of the size of the neutral segment and the length of the polycation block have been effectively exploited for assembling different PIC nanostructures [13,15]. For example, poly(ethylene glycol)-poly(L-lysine) (PEG-PLL) block copolymers having relatively large PEG segments for steric hindrance and PLL blocks with charges coordinating that of siRNA lead to the assembly of primary PICs, so-called unit PIC (uPIC), loading a single siRNA molecule and avoiding multimolecular association [15,16]. Decreasing the size of the PEG segment in the block catiomers allowed the association of uPICs into PIC vesicles (siRNAsomes), with siRNA and the cationic segments forming the vesicular membrane [13]. Moreover, adding attractive forces to the cationic block, such as conjugating hydrophobic moieties or cross-linking units, has been applied for constructing siRNA-loaded PIC micelles by promoting secondary assemblies of uPICs [12,14,17]. Notably, the structure of the RNA molecule also affects the multimolecular association of PICs. In particular, the relatively rigid structure of siRNA allowed selective formation of uPICs after mixing with PEG-PLL, while the relatively more flexible single stranded RNA (ssRNA) formed PIC micelles by compensating the restrained conformational freedom in uPICs through a segregated PIC core in multimolecular micelles [18]. Based on these observations, it is reasonable to hypothesize that the polycation flexibility may also be a contributing factor in the formation of PICs with siRNA.

Herein, we aimed to gain insight on the effect of the flexibility of the cationic segment of the block catiomers on the complexation with siRNA. Thus, to discriminate the contribution of the flexibility of the polycation, we used two corresponding block copolymers bearing polycation segments with different flexibility and PEG, as follows: PEG-PLL with the relatively rigid amide backbone, and PEG-poly(glycidyl butylamine) (PEG-PGBA) having a polyether backbone, which presents ether bonds with low rotational barrier [19,20]. The block catiomer systems have similar PEG length and weight fraction, and equivalent polycation length, i.e. 40 units (Figure 1). Moreover, the pendant primary amines serving as the cationic moieties have the same distance to the block backbone, i.e. a (CH_2_)_4_ spacer. In addition, we have previously found that PEG-PLL and PEG-PGBA have comparable degree of hydration as determined by differential scanning calorimetry (DSC) [21]. Besides, since the ionic strength and temperature affect the formation of polyion complexes [22], in our study, we have fixed these parameters for relating the differences in complexation to the polymers. The PICs resulting from mixing siRNA and PEG-PLL, or PEG-PGBA, were compared by analyzing the polymer complexation, the size and morphology of the PIC, and the PICs stability. Moreover, molecular dynamics (MD) simulations were applied to gain understanding on the siRNA-polycation interactions. In addition, the gene knockdown ability was evaluated in cancer cells to determine the in vitro delivery efficiency. Our results indicate that the flexibility of the cationic block could be an important parameter affecting PIC stability and the ensuing delivery efficiency, opening new opportunities for enhanced siRNA delivery.

## 2. Materials and methods

### 2.1. Materials

ε-Trifluoroacetyl-L-lysine N-carboxyanhydride (Lys-(TFA)-NCA) was purchased from Chuo Kaseihin Co. Inc. (Tokyo, Japan). α-Methoxy-ϵ-amino poly(ethylene glycol) (MeO-PEG-NH_2_) (*M_w_* = 12 kDa) was purchased from NOF CORPORATION (Tokyo, Japan). N,N-Dimethylformamide (DMF) (purity > 99.5%), thiourea (purity > 99.0%), hydroxylamine-O-sulfonic acid (HOSA) (purity > 90.0%) and fluorescamine were purchased from Tokyo Chemical Industry Co., Ltd. (Tokyo, Japan). Triisobutylaluminum (iBu_3_Al; 1.0 M in toluene solution), ethanol (purity > 99.8%), super dehydrated toluene (purity > 99.5%), heparin sodium and super dehydrated tetrahydrofuran (THF) (purity > 99.5%) were purchased from Fujifilm Wako Pure Chemical, Co., Inc., (Tokyo, Japan). α-Methoxy-ϵ-hydroxy poly(ethylene glycol) (MeO-PEG-OH) was purchased from Creative PEGWorks (Chapel Hill, North Carolina, USA). 4-(2-Hydroxyethyl)-1-piperazineethanesulfonic acid (HEPES) (1.0 M), 1,2-epoxy-5-henene (purity > 97.0%) and borane tetrahydrofuran complex solution 1.0 M in THF were purchased from Sigma-Aldrich (St. Louis, Missouri, USA). RNA, 5ʹ-FITC-labeled RNA, and 5ʹ-Cy5-labeled RNA were purchased from Hokkaido System Science Co., Ltd. (Hokkaido, Japan). The sequence of siRNA used is to target firefly GL3 luciferase with sense: 5′-CUU ACG CUG AGU ACU UCG AdTdT-3′ and anti-sense: 5′-UCG AAG UAC UCA GCG UAA GdTdT-3′. Dye (FITC or Cy5) was attached to the 5′ end of the sense strand. siScramble: 5ʹ-UUCUCC GAACGUGUCACGUdTdT-3ʹ (sense strand), 5ʹ-ACGUGACACGUUCGGAGAAdTdT-3ʹ (antisense strand). Alexa Fluor™ 647 NHS Ester, fetal bovine serum (FBS), Opti-MEM Reduced-Serum Medium and Dulbecco’s Modified Eagle’s Medium (DMEM) were purchased from Thermo Scientific Fisher Inc. (Waltham, Massachusetts, USA). Hela-luc cell line was purchased from Caliper LifeScience (Hopkinton, Massachusetts, USA).

### 2.2. Polymer synthesis

PEG-PLL and PEG-PGBA polymers were prepared as previously described [21,23]. The synthetic procedures are as follows: The PEG-PLL block copolymer was synthesized by ring-opening polymerization (ROP) of Lys-(TFA)-NCA. A thiourea solution was prepared by adding 1500 mg (19 mmol) in 20 mL DMF. MeO-PEG-NH_2_ (*M_n_* = 12 kDa, 0.045 mmol, 500 mg) and Lys-(TFA)-NCA (1.875 mmol, 500 mg) were dissolved with the thiourea solution. Then, the NCA solution was added to the PEG solution under argon atmosphere. The mixture was kept stirring under 35°C for 3 days, precipitated in cold ether to get *α*-methoxy-poly(ethylene glycol)-poly(N-ε-trifluoroacetyl-L-lysine) (PEG-PLL(TFA)). Deprotection of the TFA group was performed by dissolving the prepared PEG-PLL(TFA) in 1 M NaOH methanolic solution and stirred at 35°C for 12 h. After that reaction solution was dialyzed against 0.01 M HCl three times and pure water three times. The solution in the dialysis bag was lyophilized to obtain PEG-PLL. The solution in the dialysis bag was lyophilized to obtain PEG-PLL. The polymer was characterized by ^1^H-NMR analysis (D_2_O; 25°C). The degree of polymerization (DP) of the resulting PLL segment was determined to be 38 units by ^1^H-NMR by comparing the peaks of -CH_2_-CH_2_-O- (δ = 3.54 ppm) in PEG backbone and the peaks of -CH_2_-CH_2_-CH_2_-CH_2_-NH_2_ (δ = 1.10–1.80 ppm) in PLL side chain (Supplementary Figure s1).

PEG-PGBA block copolymer was synthesized by ROP and followed by brown hydroboration-amination. Briefly, MeO-PEG-OH (*M_n_* = 12 kDa, 0.0083 mmol, 100 mg) was dissolved in benzene and frozen dried under vacuum. The polymer was then dissolved in 9 mL toluene. Then, 1, 2-epoxy-5-hexene (0.415 mmol, 46.7 μL) was added to the solution and followed by addition of iBu_3_Al (11 μmol, 11 μL) under argon atmosphere. The mixture was stirred at room temperature for 24 h. The reaction was stopped by adding excess amount of ethanol (2 mL). After that, the polymer was precipitated in cold ether to obtain *α*-methoxy-poly(ethylene glycol)-poly(glycidyl butene) (PEG-PGB) block copolymer. Brown hydroboration-amination was performed by dissolving PEG-PGB (4.8 μmol, 76.2 mg) in 6.8 mL THF. Then, the borane tetrahydrofuran complex (BH_3_-THF) (2.8 mmol, 3.2 mL) was added. The mixture was stirred for 3 h. After this, HOSA (2.8 mmol, 320 mg) was added to the solution. Finally, the mixture was stirred for another 3 h, and precipitated in cold ether to obtain PEG-PGBA. The DP of PGBA segments in PEG-PGBA was determined to be 40 units from the ^1^H-NMR (D_2_O; 25°C) spectrum based on the peak intensity ratio of -O-CH3 (δ = 3.39 ppm) protons in PEG side chains to -CH2-CH2-CH2-CH_2_-NH_2_ (δ = 1.25–1.53 ppm) protons in PGBA side chain (Supplementary Figure 2). The amine in the obtained polymer was estimated from ^1^H-NMR spectrum based on the peak intensity ratio of -O-CH3 (δ = 3.39 ppm) protons in PEG side to -CH2-NH2 (δ = 2.69 ppm) in PGBA side chain (Supplementary Figure 2).

### 2.3. PIC preparation

Block copolymers and siRNA (GL3-siRNA) were separately dissolved in 10 mM HEPES buffer (pH 7.4). siRNA-loaded PICs were prepared by mixing block catiomers (1 mg/mL) and siRNA at molar ratios of primary amines in polymer to phosphates in the siRNA (N/P ratio) of 0.5, 1.0, 1.5, 2.0, 2.5 and 3.0 (final siRNA concentration around 0.015 mM). The solutions were vortexed briefly and incubated at 4°C for 1 h before use. The particle size distribution, the polydispersity index (PDI) and the derived count rate were determined by dynamic light scattering (DLS) measurement at 25°C by Zetasizer Nano ZS (Malvern Instruments Ltd, UK).

### 2.4. Determination of composition of PICs

PEG-PGBA PICs and PEG-PLL PICs solutions at N/P 0.5, 1.0, 1.5, 2.0, 2.5 and 3.0 were placed in a Vivaspin 500 protein concentrator spin columns (molecular weight cut-off (MWCO): 50,000 Da for PEG-PGBA PICs and MWCO: 30,000 Da for PEG-PLL PICs respectively; GE Healthcare, UK) and centrifuged at 5,000 rpm for 1 h to separate free polymer from siRNA associated PICs. The polymer in the ultrafiltrate was then quantified by fluorometric assay of primary amines with fluorescamine.

### 2.5. Cryogenic transmission electron microscopy (Cryo-TEM) measurement

The shape of PEG-PGBA PICs were observed by Cryo-TEM. Before measurement, PEG-PGBA PICs were cross-linked with glutaraldehyde (GA) at a residual molar ratio of GA to primary amines in PEG-PGBA ([GA]/[NH_2_]) = 2 at room temperature for 30 min. The excess GA were quenched by dialysis with 10 mM HEPES and 1 mM glycine solution for 24 h. Then, an aliquot of 3.0 μL diluted sample solution was added to negatively glow discharged carbon films grids (Cu 200 mesh), blotted to remove the excess sample solution, and plunge-frozen by an Automatic Plunge Freezer EM GP2 (Leica Microsystems, Wetzlar, Germany) with liquid ethane. The vitrified specimen was transferred to a multipurpose transmission electron microscope JEM 2100 (JEOL, Tokyo, Japan) using a cryo-holder (Model 630, Gatan, Munich, Germany). The temperature of the specimen was controlled below – 180°C throughout the analysis. TEM observation was performed at 200 kV of acceleration voltage and 10 pA/cm^2^ beam current.

### 2.6. Fluorescence correlation spectroscopy (FCS) measurement

FCS records the fluctuation of fluorescence intensity emitted from single particle during the scanning in detection volume. Then, temporally auto-correlation analysis can quantify the fluctuation to get the strength and duration, corresponding to the quantity of the fluorescence particles and their average diffusion time through the detection volume. The FCS measurement was conducted on an LSM 780 confocal laser scanning microscope (C-Apochromat, Carl Zeiss, Oberkochen, Germany) equipped with FCS function at 25°C. As the detection volume (ω) is constant during the same equipment setting, the diffusion time ($\tau_{D}$) of each sample is inversely proportional with the diffusion coefficient (D) of the particle according to Equation (1) [24–26].

(1) $$\tau_{D}=\frac{\omega^{2}}{4D}$$

Here, we used Cy5-labeled siRNA and Alexa Fluor 647-labeled polymers and performed FCS *via* HeNe laser (633 nm) scanning. The Cy5-siRNA PICs with PEG-PLL and PEG-PGBA were prepared and then diluted down to 20 nM siRNA with HEPES buffer. Diluted solutions (200 μL) were put into an 8-well Lab-Tek chamber slides (Nalgene Nunc International, Rochester, New York, USA), followed by measurement at room temperature. Compared to free siRNA, the siRNA-loaded PICs have larger hydrodynamic size, leading to lower diffusion coefficient according to Stokes-Einstein equation, and presenting larger diffusion time, as it appears in the measurement. Thus, the increase of the diffusion time of the samples refers to the assembly of free molecules into larger particles. In this experiment, the autocorrelation curve for each sample was obtained from 10 measurements at a sampling time of 10 s with the measurements being repeated 10 times. Moreover, for more accurate quantification, Alexa Fluor 647 dye was used as a standard sample for reference. As the diffusion coefficient of Alexa Fluor 647 is a known value [27], the diffusion coefficient of other samples could be calculated by comparing their diffusion time result with that of Alexa Fluor 647. Then, the hydrodynamic size of the particle can be obtained from the Stokes-Einstein equation, where $k_{B}$ is the Boltzmann constant, T is the temperature, and η is the viscosity (2).

(2) $$D_{H}=\frac{k_{B}T}{3\pi \eta D}$$

In addition, to evaluate the amount of polymers per uPIC, the polymers were labeled with Alexa Fluor 647 dye, the siRNA uPICs with Alexa Fluor 647-labeled PEG-PLL and Alexa Fluor 647-labeled PEG-PGBA were prepared at N/P = 1 and then diluted down to 20 nM siRNA with HEPES buffer. Diluted solutions (200 μL) were examined by FCS, the obtained count per molecule was analyzed to calculate the association number of polymer molecules according to the following equation:

(3) $$\begin{array}{rl} & Association\;\;number=Count\;\;per\;\;molecule\left(uPIC\right)/\\ & \;\;\;\;\;\;\;\;\;\;\;\;\;\;\;\;\;\;\;\;\;\;\;\;\;\;\;\;\;\;\;\;\;\;\;\;\;\;\;\;\;\;\;\;\;\;\;\;\;\;Count\;\;per\;\;molecule\;(fluorescence\\ & \;\;\;\;\;\;\;\;\;\;\;\;\;\;\;\;\;\;\;\;\;\;\;\;\;\;\;\;\;\;\;\;\;\;\;\;\;\;\;\;\;\;\;\;\;\;\;\;\;-labeled\;\;polymer) \end{array}$$

### 2.7. Measurement of counter polyanion exchange

The stability against counter polyanion exchange was estimated by incubating PIC solutions with heparin solution and followed by FCS measurement. Briefly, PIC solutions containing 20 pmol Cy5-siRNA were mixed with heparin sodium at different S/P ([sulfate in heparin]/[phosphate in siRNA]) ratios, and subsequently incubated for 30 min at room temperature. Next, FCS was conducted to obtain the diffusion coefficient of PIC solutions.

### 2.8. Molecular dynamic simulations of polymer/siRNA interaction

siRNA was modeled using a 19-base double strand. The RNA sequence matches the GL3 siRNA. One sequence is 5´-UCG AAG UAC UCA GCG UAA G-3´ and the other is 3´-AGC UUC AUG AGU CGC AUU C-5´. The starting coordinates were built to be a canonical A-form using AMBER NAB tool. Five polymers were analyzed, as follows: PEG with 10 repeating units; PLL with 10 repeating units; PGBA with 10 repeating units; PEG with 10 repeating units; PEG-PLL with 10 repeating units of ethylene glycol and 10 repeating units of L-lysine groups; PEG-PGBA with 10 repeating units of ethylene glycol group and 10 repeating units of glycidyl butylamine groups. The block length of 10 units was selected as a representative value to reduce computational burden. The primary amines were set with +1 charge, making the polymer charge +10 at physiological conditions. These polymers were then set to complex with siRNA in 1:1 molar ratio, which was considered to be a reliable model for exploring the details of binding based on previous reports [28]. The main characteristics of the simulated systems were summarized in Table 1.

All simulations and data analyses were performed using AMBER 18 suite of programs. The OL3 (chiOL3) force field was chosen to describe siRNA, and General Amber Force Field (GAFF) and TIP3P were used for polymers and water, respectively. Polymer was built by connecting monomer head by head, and the complex was then run *via* tleap (terminal LEap) to prepare input files for the AMBER molecular mechanics programs. Each polymer chain was placed at 10 Å from RNA at the beginning of the simulations. Polymers were initially built in the fully extended conformation, solvated in TIP3P water box under periodic boundary condition, and neutralized by adding Cl ^–^ and Na^+^ ions (salt concentration of 0.15 M). First, energy minimization was carried out on water molecules by 10,000 steps (including 5,000 steps of steepest descent and 5,000 steps of conjugate gradient) with the RNA and polymer being restricted. Next, the system was left free to move, and the whole system was minimized by 10,000 steps (including 5,000 steps of steepest descent and 5000 steps of conjugate gradient). The system was then heated from 0 k to 300 k for 50 ps, then followed by a density equilibration run (50 ps) under NPT condition (constant particle number (N), regulated pressure (P) and constant temperature(T)). The production dynamic lasted for 100 ns for each system under NPT condition. Finally, CPPTRAJ model of AMBER 18 was used to analyze the dynamic trajectories (including RMSD, RMSF and Radius of gyration), and molecular visualization was done using the Visual Molecular Dynamics (VMD) package.

Energy analysis of each system was carried out using the Molecular Mechanics Poisson-Boltzmann Surface Area (MM-PBSA) method [29], which was described through the following equation:

(4) $$\Delta {\text{G}}_{bind}\text{=}\Delta {\text{H}}_{bind}\text{-T}\Delta {\text{S}}_{bind}$$

(5) $$\Delta {\text{H}}_{bind}\text{=}\Delta {\text{E}}_{MM}\text{+}\Delta {\text{G}}_{PB}\text{+}\Delta {\text{G}}_{SA}$$

$\Delta E_{MM}=\Delta E_{bond}-\Delta E_{angle}+\Delta E_{torsion}+\Delta E_{vdw}$+$\Delta E_{ele}$ (6)

The enthalpic change in the gas phase upon RNA polymer binding (ΔE*_MM_*) was calculated by summing the bonded term (bond energy ΔE*_bond_*, angle energy ΔE*_angle_* and torsion energy ΔE*_torsion_*). ΔG*_PB/SB_* and ΔG*_SA_* depict the polar solvation energy and the nonpolar solvation energy, respectively. This part of the calculation was conducted by using the per-residue scheme implemented in MMPBSA.py script. More details could be found in AMBER 18 reference manual.

### 2.9. Cytotoxicity assay

Hela-luc cells were cultured on 96-well plates (3,000 cells per well), followed by 24 h incubation in DMEM containing 10% FBS. Next, the cells were washed twice with phosphate buffered saline (PBS), and polymers solutions were applied to each well at 0.5 mg/mL, 0.2 mg/mL, 0.1 mg/mL and 0.05 mg/mL. The cytotoxicity of the polymers was evaluated after 24 h by Cell Counting Kit-8 (CCK-8, Dojindo Molecular Technologies Inc., Tokyo, Japan) assay following manufacture’s instruction. UV absorbance was measured at 450 nm by multimode microplate reader (Tecan Group Ltd., Switzerland) to determine the cell viability.

### 2.10. Cellular uptake

Hela-luc cells (30,000 cells) were cultured on 8-well chambered borosilicate cover glass (Lab Tek) and incubated in Opti-MEM medium for 24 h. Next, naked siRNA, FITC-labeled siRNA-loaded PICs based on PEG-PLL and PEG-PGBA were applied to each well (100 nM siRNA per well). Uptake of PICs was evaluated after incubation for 24 h. Cell nucleus was stained with Hoechst for 5 min. The cells were then washed with fresh media and imaged by LSM 780 confocal laser scanning microscope.

### 2.11. In vitro gene silencing

Hela-luc cells were plated onto 96-well plates (10,000 cells per well), followed by 24 h incubation in DMEM containing 10% FBS. Next, naked GL3 siRNA, and siRNA-loaded PICs based on PEG-PLL and PEG-PGBA were applied to each well at 20, 100 and 200 nM siRNA per well. Also, a scrambled sequence (siScramble) was used to verify the sequence specificity of GL3 siRNA in each group. After 24 h, the cells were washed with 0.1 mL of PBS twice and lysed with 0.02 mL of cell culture lysis buffer (Promega, Madison, Wisconsin, USA). The luciferase activity of the lysates was determined from the photo luminescence intensity using the Luciferase Assay System (Promega, Madison, Wisconsin, USA).

### 2.12. Statistical analysis

The results are presented as mean ± standard deviation (s.d.). Groups were compared by performing Welch’s *t*-tests in Graph Pad Prism 8. P values smaller than 0.05 were considered statistically significant.

## 3. Results and discussion

### 3.1 Assembly of siRNA-loaded PICs

**Figure 2.**
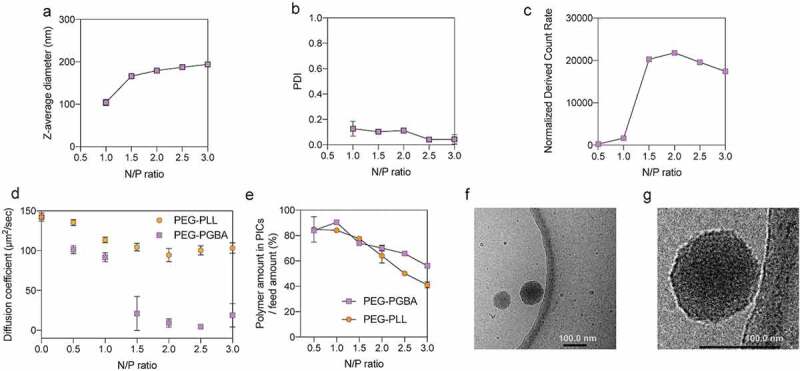
Characterization of PIC structures from PEG-PGBA and PEG-PLL. (a) Z-average diameter, (b) polydispersity index (PDI), and (c) normalized derived count rate of PEG-PGBA PICs, the final siRNA concentration was above 10 μM. The results are expressed as the mean ± s.d. (*n* = 3) for (a) to (c). (d) Diffusion coefficient of PICs from PEG-PGBA (purple square) and PEG-PLL (orange circle), the final siRNA concentration was 20 nM. (e) The amount of polymer in siRNA PICs/ feed amount. siRNA PICs were prepared from PEG-PGBA (purple square) and PEG-PLL (orange circle), the final siRNA concentration was above 10 μM. The results are expressed as the mean ± s.d. (*n* = 3). (f) Representative cryo-TEM micrograph of PEG-PGBA PICs. (g) Zoomed cryo-TEM image of PEG-PGBA PICs. Scale bars indicate 100 nm

The complexation of the block copolymers with siRNA was studied at 25°C in 10 mM HEPES buffer (pH 7.4). PEG-PGBA block copolymers were mixed with siRNA (20 μM siRNA; HEPES, PH 7.4) at varying N/P ratios. The PIC formation was followed by DLS measurement at different N/P ratios with PEG-PGBA. At N/P ratios equal to – and higher than – 1.0, the Z-average diameter (Figure 2(a)) and the PDI (Figure 2(b)) of the PEG-PGBA/siRNA mixtures were found to be less than 200 nm and lower than 0.2, respectively, indicating the formation of multimolecular PICs. In addition, the PDI of the PEG-PGBA/siRNA mixtures decreased as the N/P increased (Figure 2(b)). The PEG-PGBA/siRNA PICs at N/P = 1.0 exhibited a diameter of 104 nm and polydispersity index (PDI) of 0.13 by DLS (Figure 2(a,b)), though the normalized derived count rate for this sample was still quite low, suggesting a small amount of multimolecular PICs being formed in the solution. With further increasing N/P from 1.0 to 1.5, the size of the PEG-PGBA/siRNA PICs increased from 104 to 166 nm and reached a plateau, while maintaining the initial PDI. The normalized derived count rate, which is related to the particle size and the concentration of particles, also increased, supporting the formation of larger particles. At N/P equal to – and higher than – 1.5, the normalized derived count rate of PEG-PGBA PICs was kept at comparable level (Figure 2(c)), which indicates the formation of large particles at comparable concentration. It is worth noting the formation of 200 nm PEG-PGBA PICs with PDI of around 0.2 at N/P equal to – and higher than – 0.5 was also found in 10 mM HEPES with 150 mM NaCl (Supplementary Figure 3).

Because the size of PEG-PLL PICs is too small for precise assessment by DLS with Zetasizer even at the polymer concentration of 10 mg/mL, we then studied the complexation behavior of siRNA with PEG-PLL and PEG-PGBA by FCS. In the FCS measurement, fluorescence is used for recording the diffusion time of molecules. Thus, Cy5-labeled siRNA was used at a diluted concentration of 20 nM to avoid saturation of the fluorescence signal. Accordingly, the changes in D of Cy5-siRNA were measured by FCS after mixing with the catiomers at increasing N/P ratio, while keeping the total concentration of anionic and cationic residues constant to avoid changes in the ionic strength [30]. In the complexation of PEG-PLL and Cy5-siRNA, D decreased from 142 ± 5 μm^2^/s of free Cy5-siRNA to 113 ± 4 μm^2^/s of uPICs with the increasing of N/P above 1 (Figure 2(d)). Adding more PEG-PLL to siRNA did not induce significant changes in D, which supports the steady formation of uPICs as observed in previous reports [18]. In addition, the hydrodynamic diameter of PEG-PLL uPICs was calculated based on diffusion coefficient obtained from the FCS measurements (Supplementary Figure 5). The results showed that the uPICs of PEG-PLL/siRNA at N/P equal to – and higher than – 1 are approximately 7 nm, which is consistent with the previously reported diameter of 8.7 nm [18]. In the case of PEG-PGBA, the diffusion coefficient decreased to the level of uPIC with increasing of N/P to 1. It is worth noting that the different siRNA concentrations in the DLS and FCS measurements affected the formation of PEG-PGBA/siRNA PICs at N/P = 1.0. Thus, the PEG-PGBA/siRNA PICs exhibited a diameter of 104 nm and polydispersity index (PDI) of 0.13 by DLS (Figure 2(a,b)), though the normalized derived count rate for this sample was still quite low, suggesting a small amount of multimolecular PICs being formed in the solution. These multimolecular PICs of PEG-PGBA/siRNA at N/P = 1.0 observed in DLS at 10 μM are not stable upon dilution to 20 nM, becoming uPICs as indicated by the FCS results. Both PEG-PGBA/siRNA and PEG-PLL/siRNA PICs were also found to have comparable levels of diffusion coefficient at N/P = 1 by FCS, indicating the formation of uPICs for both polymers.

To evaluate the number of polymer molecules per uPICs, the polymers were labeled with Alexa Fluor 647 dye, and the association number of fluorescence-labeled polymer per uPIC at N/P = 1 was examined by FCS. Accordingly, the results showed that both PEG-PGBA/siRNA and PEG-PLL/siRNA have around 1 polymer per uPIC (Supplementary Table s1). Thus, we can assume PEG-PGBA/siRNA and PEG-PLL/siRNA have a similar PEG density. At N/P = 1.5 or higher, the large multimolecular PICs of PEG-PGBA/siRNA are detectable in both DLS and FCS. The rising of the N/P to 1.5 for PEG-PGBA dramatically decreased D to 9 ± 5 μm^2^/s, suggesting secondary association of uPICs into multimolecular PICs (Figure 2(d)). These observations correlate with the DLS results at 20 nM siRNA, where the formation of 200 nm particles with PDI of around 0.2 and occurred at N/P equal to – and higher than – 1.5 (Supplementary Figure 4), suggesting the ability of PEG-PGBA to promote secondary associations. The amount of PEG-PGBA and PEG-PLL in the PICs was quantified by removing free polymers by ultrafiltration and evaluating the amount of free polymers in the ultrafiltrate by fluorescamine method. The results showed that most PEG-PGBA and PEG-PLL were effectively associated into PICs during PIC formation (Figure 2(e)). For PEG-PLL, increasing the amount of polymer did not augment the number of polymer molecules associated to siRNA, keeping close to the stoichiometric ratio N/P = 1 (Supplementary Figure 6), which correlates with the formation of uPICs at all the studied N/P ratios (Figure 2(d)). For PEG-PGBA, the amount of polymers in the PICs increased with the N/P ratio (Supplementary Figure 6), supporting the formation of nonstoichiometric structures with more polymer than siRNA. These observations of PEG-PGBA are consistent with the formation of multimolecular PICs in the N/P range higher than unity, and in contrast with the result obtained for PEG-PLL, which keeps the stoichiometric uPIC structure even at N/P ≥ 1.

Then, direct observation of the shape and size of PICs from PEG-PGBA at N/P = 2 was conducted by cryo-TEM (Figure 2(f,g)). Cryo-TEM images showed spherical shapes of multimolecular PICs with a macromolecule-rich phase inside the particles of around 127 ± 25 nm diameter (as determined from 50 PEG-PGBA PICs). The assembling process of PEG-PGBA multimolecular PICs could be explained from the thermodynamic standpoint. Multimolecular PICs formation is facilitated by the reduction of interfacial free energy from the phase separation between aqueous phase and PIC moiety [31,32]. Moreover, the increment of conformational freedom (entropy gain) of charged segments in the segregated phase of PICs contributes to the formation of multimolecular PICs [31,32]. On the other hand, the steric repulsion between the neighboring PEG strands in the shell layer of multimolecular PICs can be an impeding factor [31–33]. Thus, in the PEG-PLL/siRNA system, because of relatively rigid structure, the increment of conformational freedom in a multimolecular PIC phase may not be enough to compensate with the steric repulsive effect of PEG strands, remaining as uPIC after complexation [18]. Notably, when PEG-PLL was complexed with a flexible single-stranded RNA (ssRNA), core-shell polymeric micelles were obtained [18]. The higher flexibility of ssRNA ensured a higher entropy at the initial state, while the complexation of single stranded RNA with PEG-PLL results in restrained conformational freedom in uPIC of PEG-PLL and ssRNA [18]. This loss of entropy gain could be compensated through multimolecular micelle formation, as the ssRNA gets higher conformational freedom in the micelle core [18]. In the PEG-PGBA/siRNA system, the chain conformational entropy loss resulting from the polycation chain can be compensated by association of PEG-PGBA uPICs into multimolecular PICs, as the PGBA strands could get a higher degree of conformational freedom within the segregated phase of PICs compared to the uPIC state. As we discussed above, the multimolecular PICs formation is based on the balance between interfacial energy and conformational entropy of the polymer strands, and the steric repulsion of the PEG shell. Probably, the driving force in the PEG-PGBA system may still not be enough for the formation of a spherical core-shell micelle, resulting in relatively larger PICs to compensate the steric repulsion from the PEG strands.

### 3.2 Stability of PICs

**Figure 3.**
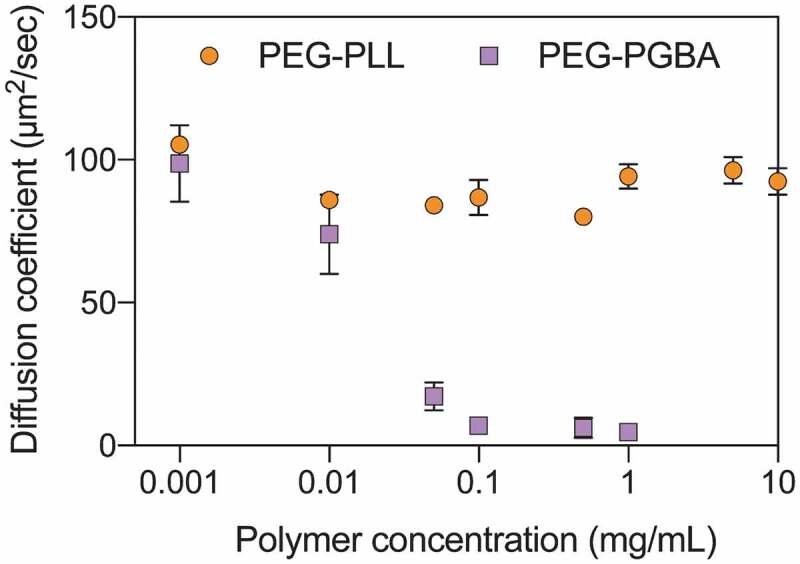
Diffusion coefficient of Cy5-labeled siRNA after mixing with different polymer concentrations of PEG-PLL (orange circle) and PEG-PGBA (purple square). The N/P ratio of each group was kept at 2. All results are expressed as the mean ± s.d. (*n* = 10)

To further understand the influence of the catiomer structure, we studied the stability of the PICs formed with PEG-PLL and PEG-PGBA. Thus, the critical association concentration (CAC) of the PICs was evaluated by preparing PICs loading Cy5-siRNA with increasing polymer concentrations (0.005 mg/mL–10 mg/mL) at a constant N/P of 2. Then, the changes in the diffusion coefficient of Cy5-siRNA were calculated from the diffusion times obtained by FCS. For PEG-PLL, the changes in D of Cy5-siRNA indicated that the catiomer formed uPICs at the lowest studied concentration of 0.001 mg/mL and avoided multimolecular association even at high polymer concentration (10 mg/mL) (Figure 3). When Cy5-siRNA was mixed with PEG-PGBA, the changes in D showed that it formed uPICs at 0.001 mg/mL, followed by a remarkable drop in D at concentrations higher than 0.05 mg/mL (Figure 3), which can be related to multimolecular assembly above a CAC, as previously observed for micellar and vesicular PICs [18,34]. Thus, these results suggest that the flexible PEG-PGBA promotes the association of uPICs into multimolecular assemblies, with a CAC of around 0.05 mg/mL at N/P = 2.

**Figure 4.**
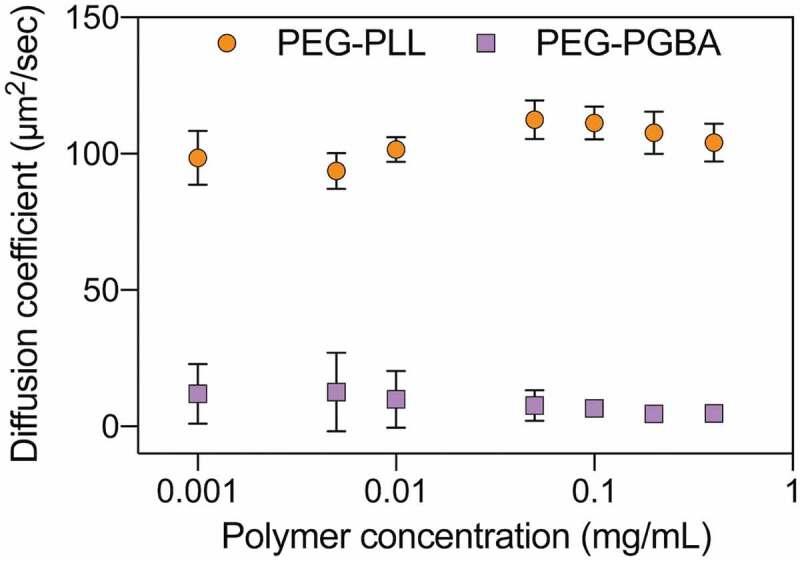
Diffusion coefficient of PEG-PLL uPICs (orange circle) and PEG-PGBA PICs (purple square) loading Cy5-labeled siRNA (N/P = 2) after serial dilution in 10 mM HEPES. All results are expressed as the mean ± s.d. (*n* = 10)

**Figure 5.**
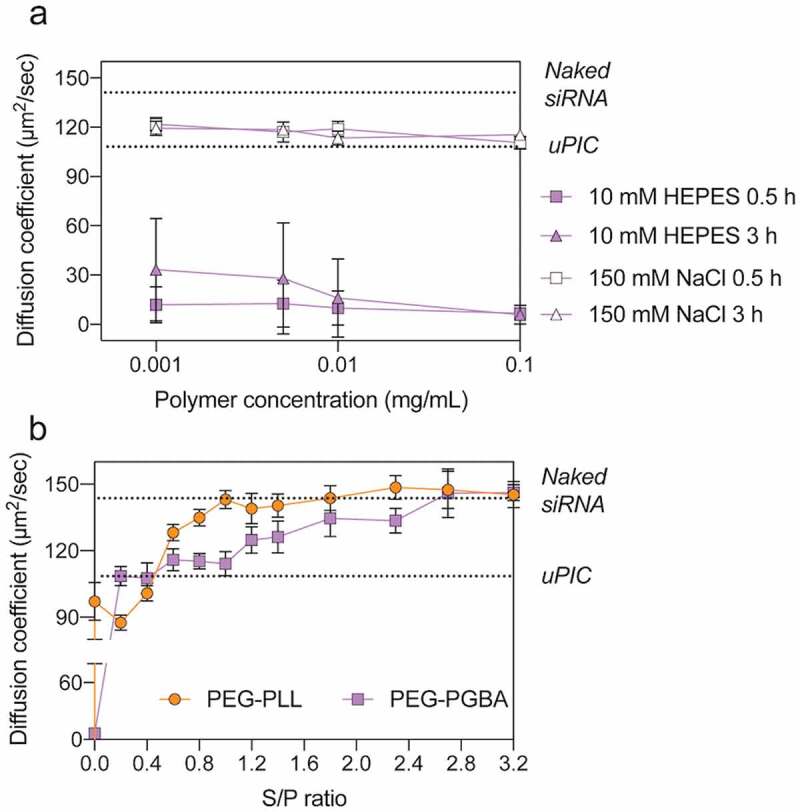
(a) Diffusion coefficient of PEG-PLL uPICs and PEG-PGBA PICs loading Cy5-labeled siRNA (N/P = 2) serially diluted with 10 mM HEPES or 150 mM NaCl and incubated for 0.5 and 3 h. (b) Diffusion coefficient of PEG-PLL uPICs (orange circle) and PEG-PGBA PICs (purple square) loading Cy5-labeled siRNA (N/P = 2) after incubation with heparin sulfate at different [sulfate in heparin]/[phosphate in siRNA] (S/P) ratios. The lower dashed line and the upper dashed line indicate the diffusion coefficient of uPIC and naked siRNA, respectively. All results are expressed as the mean ± s.d. (*n* = 10)

The dissociation of the PICs loading Cy5-siRNA was also evaluated by measuring the D values after diluting the samples (Figure 4). PEG-PLL maintained the D value of the primary uPIC assembly with Cy5-siRNA in all the tested concentrations down to 0.001 mg/mL in 10 mM HEPES buffer. When the PEG-PGBA PICs were diluted in 10 mM HEPES, the diffusion coefficient values also remained constant after incubation for 0.5 h, even at 0.001 mg/mL, indicating that PEG-PGBA kept the multimolecular association with Cy5-siRNA (Figure 4). Moreover, increasing incubation time up to 3 h did not cause large changes in D when the PEG-PGBA/siRNA PICs were diluted in 10 mM HEPES. This result suggests that the PEG-PGBA multimolecular assemblies may involve a hysteresis behavior against dilution. Such hysteresis has been previously observed in other PIC systems, such as in PICs made from PLL homopolymer and PEG-poly(L-glutamate) [35]. On the other hand, the PEG-PGBA multimolecular PICs disassembled into uPICs within 0.5 h when diluted to 0.1 mg/mL in 10 mM HEPES buffer with 150 mM NaCl (Figure 5(a)). This behavior suggests that PEG-PGBA multimolecular PICs are likely to exist predominantly in uPIC state in physiological condition.

As counter polyanion exchange from charged polysaccharides with PICs is the main mechanism for PIC dissociation in biological conditions [36,37], we therefore investigated the stability of the PICs in the presence of heparin. The PICs were incubated with heparin at different S/P ([sulfate in heparin]/[phosphate in siRNA]) ratios for 0.5 h, and the D of Cy5-siRNA was measured by FCS. The results showed that PEG-PGBA multimolecular PICs rapidly dissociated into uPICs when incubated with heparin. However, the uPICs from PEG-PGBA were able to retain siRNA at higher heparin concentrations than uPICs from PEG-PLL. The siRNAs are released from uPIC at S/P = 1 for PEG-PLL uPICs, suggesting a fast and quantitative exchange. On the other hand, excess heparin is required (S/P = 2.8) to kick out all the siRNAs from PEG-PGBA uPIC (Figure 5(b)).

### 3.3 Molecular dynamic simulations of polymer/siRNA interaction

MD simulation is a valuable tool for gaining understanding of the interaction of polymers and nucleic acids. For example, MD simulation has been successfully applied for addressing the mechanisms promoting the stability of PIC-based systems loading pDNA [38]. Thus, here we conducted MD simulations for improving our understanding on the effect of PEG-polycation flexibility on polymer/siRNA complex formation by paying particular attention to the interactions of the polycation blocks with siRNA. This MD simulation could be useful for assessing the stability of uPICs, but not for multimolecular assemblies, where entropical factors play important roles. Thus, we first monitored and compared the interactions of homo-polycation PGBA and PLL with siRNA in a 1:1 molar ratio to explore the details of binding. In this study, we used OL3 (ff99bsc0χOL3) force field for RNA in AMBER, which can remove destabilization and prevent formation of spurious ladder-like structural distortions during RNA simulations [39,40]. To build a suitable binding model, we first evaluated the influence of polycation siRNA distance. PGBA was placed at 10 Å and 20 Å from the siRNA center of mass at the beginning of the simulations. The calculation of the root mean square deviation (RMSD) was carried out to analyze the changes in structure and dynamics of the polycation siRNA complex. From the RMSD changing shown in Supplementary Figure 7a, both PGBA/siRNA and PLL/siRNA systems were found to equilibrate after 25 ns. The equilibrations were also obtained until the end of the simulations. To better understand these models, we monitored the radius of gyration (*R*_g_) over the entire trajectories. *R*_g_ is the root mean square distance of each atom from the center of mass of the structure considered, as defined by:

(4) $$\;{R_{g}}^{2}=\frac{1}{M}\sum_{i=1}^{N}m_{i}{\left(r_{i}-r_{mean}\right)}^{2}$$

*R*_g_ has been used as an indicator of the structure compactness [41]. The results showed that increasing the distance of polycation and siRNA from 10 to 20 Å has little effect on the complex conformation (Supplementary Figure 8a and Supplementary Table 2). Therefore, to save computational cost, the simulation with the polycation and siRNA with distance of 10 Å was used in the following studies.

**Figure 6.**
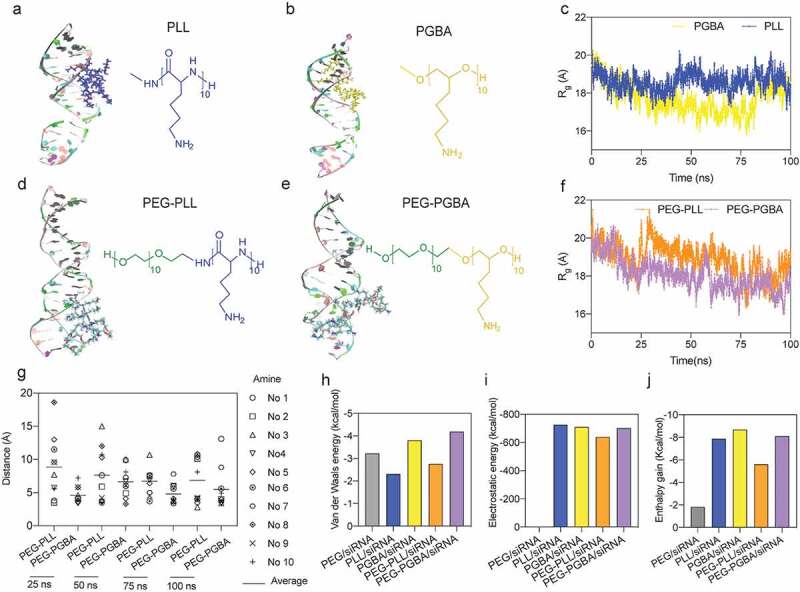
Molecular dynamics (MD) simulation of siRNA binding with polycations and block catiomers. (a), (b) Front view snapshots taken from the dynamic simulation of PLL-siRNA and PGBA-siRNA. (c) Time evolution of Radius of gyration (*R*_g_) of PLL-siRNA and PGBA-siRNA. (d), (e) Front view snapshots taken from the dynamic simulation of PEG-PLL-siRNA and PEG-PGBA-siRNA. (f) Time evolution of *R*_g_ of PEG-PLL-siRNA and PEG-PGBA-siRNA. (g) Amine/phosphate distance in PEG-PLL-siRNA and PEG-PGBA-siRNA, each amine is identified by residue numbers (No.). (h) Van der Waals energy, (i) electrostatic energy and (j) enthalpy gain during polymers/siRNA interaction (normalized per charged amine, expressed in kcal mol^–1^)

To assess the effect of polycationic flexibility on the interaction with siRNA, we first compared the interactions of the homo-polycations PLL and PGBA with siRNA at 1:1 ratio. The front view snapshots taken from the dynamic simulation of PLL/siRNA and PGBA/siRNA are shown in Figure 6(a,b), respectively. *R*_g_ of PLL/siRNA was found to be almost constant for all the solution phase simulation, while PGBA/siRNA showed a lower *R*_g_ compared with PLL/siRNA during the simulation trajectories (Figure 6(c)), suggesting a tighter complexation. To allow direct comparison between the systems, the binding energies were normalized per amine and expressed in kcal mol^−1^ (Figure 6(h,i,j)). The electrostatic energies (ΔE*_ele_*), which originated from the interaction of positive charges on the primary amines of the polycation and the negative charges on the phosphate groups of RNA, are markedly favored in both PLL/siRNA and PGBA/siRNA systems. A favorable contribution also appears from van der Waals energy (ΔE*_vdm_*), which primarily came from contacts between the polycation and siRNA. Moreover, there seems to be a slightly favorable role from nonpolar contribution to solvation energy (ΔE*_SUFR_*). On the other hand, the polar term of the solvation energy (ΔE*_GB_*) was unfavorable. The enthalpy of binding (ΔH*_bind_*) was also computed as the sum of changes of the ΔE*_ele_*, ΔE*_vdm_*, ΔE*_SUFR_* and ΔE*_GB_*. For lower ΔH*_bind_* value, there was more enthalpy gain during the polymer-siRNA binding process. Further details on the calculations are given in supporting information (Supplementary Table 3). When comparing the energy contribution during complexation of PLL/siRNA and PGBA/siRNA, it was observed that PGBA/siRNA resulted in higher van der Waals contribution and more enthalpy gain. Together with the *R*_g_ changing, these findings suggest polycation flexibility plays a role in siRNA binding.

To explore the effect of polycation flexibility on the binding of block catiomers with siRNA, we further built simulations for PEG-PLL/siRNA and PEG-PGBA/siRNA systems (Figure 6(d,e)). Moreover, PEG homopolymer without charge was analyzed and used as a negative control. To test the model, we placed PEG-PLL at opposite direction in the simulation box to assess the effect of PEG-polycationic position in the interaction. As shown in Supplementary Figure 8b and Supplementary Table 2, the binding trend of PEG-PLL/siRNA and the inverted PEG-PLL/siRNA was comparable, which supports the validity of the simulation system. The results of the model for PEG showed the negligible ΔE*_ele_* in PEG/siRNA as compared with PEG-PLL/siRNA and PEG-PGBA/siRNA, substantiating the role of electrostatic interaction in bringing the block catiomers and siRNA together (Supplementary Table 3). Moreover, PEG-PGBA showed a comparable *R*_g_ to that of PGBA, while PEG-PLL underwent a significant reorganization, resulting in a higher *R*_g_ than that of PLL (Figure 6(c,f), and Supplementary Figures 8c and 8d). These results suggested a structural restraint from PEG on the siRNA binding in the PEG-PLL/siRNA system. Thus, PEG-PGBA/siRNA presented a lower *R*_g_ compared with PEG-PLL/siRNA (Figure 6(f)). To further understand the interaction of the block catiomers with siRNA, we analyzed the average distance between the amines in the polymers and the contacting phosphates of siRNA. The results indicated the amines of PEG-PGBA were closer to the phosphates of siRNA than the amines of PEG-PLL (Figure 6(g)). In addition, the binding energies showed that PEG-PGBA/siRNA presented higher ΔE*_vdm_*, ΔE*_ele_* and enthalpy gain compared to PEG-PLL/siRNA (Figure 6(h,i,j)), which suggest a lower accessible surface area (ASA) for PEG-PGBA/siRNA complexes than for PEG-PLL/siRNA complexes. Importantly, lower ASA has been found to correlate with higher stability of PIC-based nanostructures [42], with triblock copolymer-based PICs having lower ASA allowing more efficient protection of pDNA. In this study, the more favorable binding with siRNA for PEG-PGBA than for PEG-PLL in the MD simulations is compatible with our recent findings on the binding of PEG-PGBA and PEG-PLL with mRNA, where PEG-PGBA allowed more than 50-fold stronger binding to mRNA than PEG-PLL, as determined by isothermal calorimetry analysis [21].

### 3.4 In vitro activity

**Figure 7.**
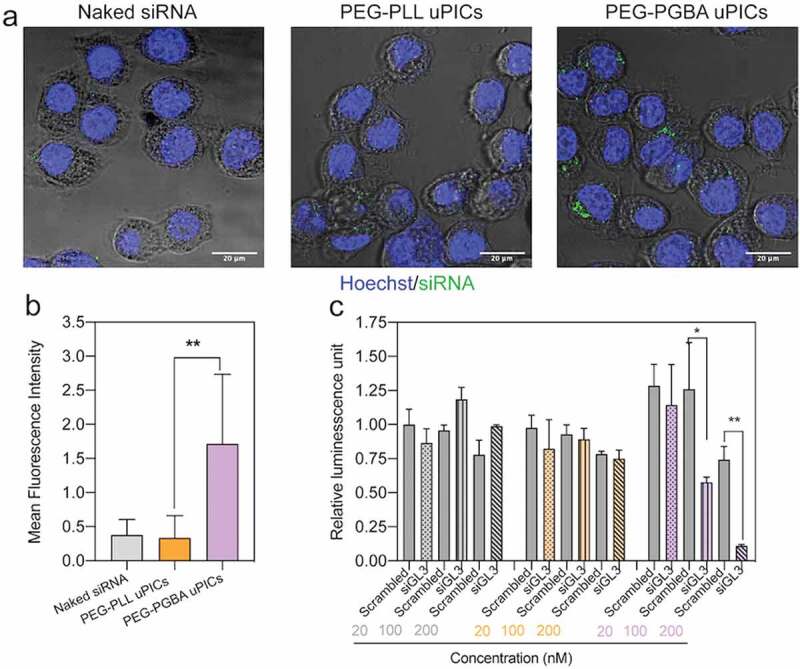
*In vitro* performance of PEG-PLL uPICs and PEG-PGBA uPICs. (a) Cellular uptake efficiency in cultured Hela-luc cells incubated for 24 h with naked siRNA, PEG-PLL uPICs and PEG-PGBA uPICs. Scale bars = 20 μm. (b) Quantification of cellular uptake measured by the mean fluorescence intensity of the pixels corresponding to FITC. Data represent the mean ± s.d. (*n* = 20 cells). (c) Gene silencing efficiency of naked siRNA (gray column), PEG-PLL uPICs (orange column) and PEG-PGBA uPICs (purple column) loaded with scrambled siRNA or siGL3 at 20, 100 and 200 nM siRNA in cultured Hela-luc cells after 24 h. Data represent the means ± s.d. (*n* = 4). *p* < 0.05 (*) and *p* < 0.01 (**) were calculated by the Welch’s t-test

According to the stability tests, PEG-PGBA multimolecular PICs are disrupted into uPICs by physiological salt concentration and heparin. Thus, the *in vitro* activity evaluation of PEG-PLL- and PEG-PGBA-based systems was compared between PEG-PGBA uPICs and PEG-PLL uPICs. First, we confirmed the safety of the catiomers by studying the *in vitro* cytotoxicity. After incubating Hela-luc cells with the polymers for 24 h, we found both PEG-PLL and PEG-PGBA are noncytotoxic at concentrations lower than 0.05 mg mL^−1^ (Supplementary Figure 9). Thus, concentrations lower than 0.05 mg mL^−1^ were used in the subsequent cellular studies. The cellular uptake was studied in Hela-luc cells by using FITC-labeled siRNA and CLSM. Cells were treated with 100 nM siRNA, the final polymer concentration was calculated to be 0.0021 mg/mL and 0.0015 mg/mL for PEG-PLL and PEG-PGBA, respectively. After 24 h incubation, we found that the fluorescence intensity of the FITC signal in the cells treated with PEG-PGBA uPICs was fivefold higher than that of PEG-PLL uPICs (Figure 7(b)). Generally, PEG shielding reduces the internalization of PEGylated nanomedicines into cells, so called ‘PEG dilemma’ [43]. However, in the case of negatively charged free siRNA, the cellular uptake is even lower due to the electrostatic repulsion with the negatively charged cellular surface. Thus, the charge neutralization of siRNA by PIC formation can improve the intracellular access, the siRNA loaded in the PICs can be delivered into the cytoplasm after a relatively long incubation [10], as also indicated in our study. Importantly, cells present negatively charged polysaccharide chains on their surface that act as a barrier for the internalization of carriers based on PIC assembly by promoting the dissociation of the PIC complexes before cellular uptake [44]. In our study, the stability of the PICs against heparin indicates that PEG-PGBA allowed higher stability against the counter polyanion exchange than PEG-PLL (Figure 5(b)). Moreover, the simulation results showed that PEG-PGBA presents a tighter association with siRNA compared with the rigid PEG-PLL. Thus, it is reasonable to assume that PEG-PGBA PICs with tight complexation with siRNA could perform better against dissociation induced by polyanions on the cell surface than PEG-PLL PICs, resulting in a higher cellular uptake.

Finally, the ability of uPICs to knockdown luciferase was also determined in Hela-luc cells. After treating the cells with 20 nM–200 nM siRNA, the final polymer concentration was calculated to be 0.00030 mg/mL to 0.0060 mg/mL for PEG-PGBA. According to the stability results in 150 mM NaCl (Figure 5(a)), PEG-PGBA multimolecular PICs are in uPIC state at these concentrations. As shown in Figure 7(c), the cells treated with PEG-PGBA uPICs showed a dose dependent gene knockdown, with a modest (∼54%) and high gene decrease (~85%) at 100 and 200 nM GL3 siRNA, respectively. On the other hand, naked siRNA and PEG-PLL uPICs showed negligible luciferase knockdown. These results revealed that PEG-PGBA uPICs improved the gene silencing *in vitro*, presumably due to the higher intracellular delivery. It is also important to note that PEG-PLL and PEG-PGBA have the same PEG segment and similar polycation length. Moreover, we have previously reported that the degree of hydration of PEG-PLL and PEG-PGBA is comparable [21]. Thus, presumably, the difference in these two systems may chiefly be due to the different backbone structure of the polycation, *i.e*. the peptide bonds in PEG-PLL and the ether bonds in PEG-PGBA. The results from polyanion exchange showed the uPIC from PEG-PGBA is more stable than the uPIC from PEG-PLL (Figure 5(b)). The molecular simulations also indicate tighter siRNA complexation for PEG-PGBA than for PEG-PLL. In addition, we have shown that PEG-PGBA can increase the affinity to RNA by 50-fold compared to PEG-PLL [21]. Thus, it is safe to conclude that the tighter binding of PEG-PGBA to siRNA compared to PEG-PLL in uPIC plays a substantial role in the difference in the intracellular delivery and knockdown efficiency observed in this study.

## 4. Conclusion

Our results demonstrated that PEG-polycation chain flexibility is an important factor in siRNA PIC formation. PEG-PLL with a relatively rigid amide bond allowed the exclusive formation of uPICs even at high polymer concentrations, while PEG-PGBA with the relatively more flexible polyether backbone facilitated the multimolecular PICs formation. The multimolecular PICs were stable in salt-free solutions, even in diluted conditions, but rapidly dissociated into uPICs at 150 mM NaCl. Moreover, the uPICs from PEG-PGBA were more stable against the counter polyanion exchange than the uPICs from PEG-PLL, which may be related to the tighter complexation of PEG-PGBA with siRNA compared to that of PEG-PLL, as suggested from computational modeling studies. In *in vitro* cellular studies, PEG-PGBA uPICs allowed higher intracellular delivery of siRNA than PEG-PLL uPICs for achieving enhanced gene knockdown. Our findings indicate the implications of catiomer flexibility on PIC formation with siRNA. Furthermore, this study evolves new engineering parameters that can be applied for generating not only novel carriers with enhanced delivery efficacy, but also new biomaterial applications based on engineered polymer-RNA interactions.

## Supplementary Material

Supplemental Material

## References

[1] Davidson BL, McCray PB Jr. Current prospects for RNA interference-based therapies. Nat Rev Genet. 2011 May;12(5):329–340. 21499294 10.1038/nrg2968 PMC7097665

[2] Kim DH, Rossi JJ. Strategies for silencing human disease using RNA interference. Nat Rev Genet. 2007 Mar;8(3):173–184. 17304245 10.1038/nrg2006

[3] Elbashir SM, Harborth J, Lendeckel W, et al. Duplexes of 21-nucleotide RNAs mediate RNA interference in cultured mammalian cells. Nature. 2001 May 24;411(6836):494–498. 11373684 10.1038/35078107

[4] Rana TM. Illuminating the silence: understanding the structure and function of small RNAs. Nat Rev Mol Cell Biol. 2007 Jan;8(1):23–36. 17183358 10.1038/nrm2085

[5] Whitehead KA, Langer R, Anderson DG. Knocking down barriers: advances in siRNA delivery. Nat Rev Drug Discov. 2009 Feb;8(2):129–138. 19180106 10.1038/nrd2742 PMC7097568

[6] Aagaard L, Rossi JJ. RNAi therapeutics: principles, prospects and challenges. Adv Drug Deliv Rev. 2007 Mar 30;59(2–3):75–86. 17449137 10.1016/j.addr.2007.03.005 PMC1978219

[7] Hu B, Zhong L, Weng Y, et al. Therapeutic siRNA: state of the art. Signal Transduct Target Ther. 2020 Jun 19;5(1):101. 32561705 10.1038/s41392-020-0207-x PMC7305320

[8] Kaczmarek JC, Kowalski PS, Anderson DG. Advances in the delivery of RNA therapeutics: from concept to clinical reality. Genome Med. 2017 Jun 27;9(1):60. 28655327 10.1186/s13073-017-0450-0 PMC5485616

[9] Cabral H, Miyata K, Osada K, et al. Block copolymer micelles in nanomedicine applications. Chem Rev. 2018 Jul 25;118(14):6844–6892. 29957926 10.1021/acs.chemrev.8b00199

[10] Kim HJ, Kim A, Miyata K, et al. Recent progress in development of siRNA delivery vehicles for cancer therapy. Adv Drug Deliv Rev. 2016 Sep;1(104):61–77. 10.1016/j.addr.2016.06.011 27352638

[11] Peng L, Wagner E. Polymeric carriers for nucleic acid delivery: current designs and future directions. Biomacromolecules. 2019 Oct 14;20(10):3613–3626. 31497946 10.1021/acs.biomac.9b00999

[12] Christie RJ, Miyata K, Matsumoto Y, et al. Effect of polymer structure on micelles formed between siRNA and cationic block copolymer comprising thiols and amidines. Biomacromolecules. 2011 Sep 12;12(9):3174–3185. 21863796 10.1021/bm2006714

[13] Kim BS, Chuanoi S, Suma T, et al. Self-assembly of siRNA/PEG-b-catiomer at integer molar ratio into 100 nm-sized vesicular polyion complexes (siRNAsomes) for RNAi and codelivery of cargo macromolecules. J Am Chem Soc. 2019 Feb 27;141(8):3699–3709. 30729777 10.1021/jacs.8b13641

[14] Kim HJ, Ishii T, Zheng M, et al. Multifunctional polyion complex micelle featuring enhanced stability, targetability, and endosome escapability for systemic siRNA delivery to subcutaneous model of lung cancer. Drug Deliv Transl Res. 2014 Feb;4(1):50–60. 25786617 10.1007/s13346-013-0175-6

[15] Watanabe S, Hayashi K, Toh K, et al. In vivo rendezvous of small nucleic acid drugs with charge-matched block catiomers to target cancers. Nat Commun. 2019 Apr 24;10(1):1894. 31019193 10.1038/s41467-019-09856-w PMC6482185

[16] Kim BS, Osawa S, Yum J, et al. Installation of a thermoswitchable hydrophobic domain into a unimer polyion complex for enhanced cellular uptake of siRNA. Bioconjugate Chem. 2020 May;31(5):1320–1326. 10.1021/acs.bioconjchem.0c00238 32352276

[17] Kim HJ, Takemoto H, Yi Y, et al. Precise engineering of siRNA delivery vehicles to tumors using polyion complexes and gold nanoparticles. Acs Nano. 2014 Sep;8(9):8979–8991. 25133608 10.1021/nn502125h

[18] Hayashi K, Chaya H, Fukushima S, et al. Influence of RNA strand rigidity on polyion complex formation with block catiomers. Macromol Rapid Commun. 2016 Mar;37(6):486–493. 26765970 10.1002/marc.201500661

[19] Kang YK, No KT, Scheraga HA. Intrinsic torsional potential parameters for conformational analysis of peptides and proteins. J Phys Chem. 1996 Jan 1;100(38):15588–15598.

[20] Lowe JP. Barriers to internal rotation about single bonds. Progress Phys Oganic Chem. 1968;6:1–9.

[21] Miyazaki T, Uchida S, Nagatoishi S, et al. Polymeric nanocarriers with controlled chain flexibility boost mRNA delivery in vivo through enhanced structural fastening. Adv Healthc Mater. 2020 Aug;9(16):e2000538. 32583633 10.1002/adhm.202000538

[22] Wibowo A, Osada K, Matsuda H, et al. Morphology control in water of polyion complex nanoarchitectures of double-hydrophilic charged block copolymers through composition tuning and thermal treatment. Macromolecules. 2014 May 13;47(9):3086–3092.

[23] Kataoka K, Togawa H, Harada A, et al. Spontaneous formation of polyion complex micelles with narrow distribution from antisense oligonucleotide and cationic block copolymer in physiological saline. Macromolecules. 1996 Jan 1;29(26):8556–8557.

[24] Elson EL. Fluorescence correlation spectroscopy: past, present, future. Biophys J. 2011 Dec 21;101(12):2855–2870. 22208184 10.1016/j.bpj.2011.11.012 PMC3244056

[25] Petrov EP, Schwille P. State of the art and novel trends in fluorescence correlation spectroscopy. Springer Ser Fluores. 2008;06:145–197.

[26] Schwille P, Heinze KG, Koltermann A. Two-photon fluorescence cross-correlation spectroscopy. Biophys J. 2001 Jan;80(1):364a–364a. 10.1002/1439-7641(20010518)2:5<269::AID-CPHC269>3.0.CO;2-Y 23696501

[27] Moparthi SB, Thieulin-Pardo G, Mansuelle P, et al. Conformational modulation and hydrodynamic radii of CP12 protein and its complexes probed by fluorescence correlation spectroscopy. FEBS J. 2014 Jul;281(14):3206–3217. 24863370 10.1111/febs.12854

[28] Pavan GM, Danani A, Pricl S, et al. Modeling the multivalent recognition between dendritic molecules and DNA: understanding how ligand “sacrifice” and screening can enhance binding. J Am Chem Soc. 2009 Jul 22;131(28):9686–9694. 19555062 10.1021/ja901174k

[29] Miller BR 3rd, McGee TD Jr., Swails JM, et al. MMPBSA.py: an efficient program for end-state free energy calculations. J Chem Theory Comput. 2012 Sep 11;8(9):3314–3321. 26605738 10.1021/ct300418h

[30] Harada A, Kataoka K. Formation of polyion complex micelles in an aqueous milieu from a pair of oppositely-charged block copolymers with poly(ethylene glycol) segments. Macromolecules. 1995 Jul 1;28(15):5294–5299.

[31] Harada A, Kataoka K. Selection between block- and homo-polyelectrolytes through polyion complex formation in aqueous medium. Soft Matter. 2007 Dec 11;4(1):162–167. 32907096 10.1039/b713853a

[32] Kataoka K, Harada A, Nagasaki Y. Block copolymer micelles for drug delivery: design, characterization and biological significance. Adv Drug Deliv Rev. 2001 Mar 23;47(1):113–131. 11251249 10.1016/s0169-409x(00)00124-1

[33] Harada A, Kataoka K. Chain length recognition: core-shell supramolecular assembly from oppositely charged block copolymers. Science. 1999 Jan 1;283(5398):65–67. 9872741 10.1126/science.283.5398.65

[34] Anraku Y, Kishimura A, Yamasaki Y, et al. Living unimodal growth of polyion complex vesicles via two-dimensional supramolecular polymerization. J Am Chem Soc. 2013 Jan 30;135(4):1423–1429. 23289646 10.1021/ja3096587

[35] Mutaf OF, Kishimura A, Mochida Y, et al. Induction of secondary structure through micellization of an oppositely charged pair of homochiral block- and homopolypeptides in an aqueous medium. Macromol Rapid Commun. 2015 Nov;36(22):1958–1964. 26296388 10.1002/marc.201500368

[36] Zuckerman JE, Choi CH, Han H, et al. Polycation-siRNA nanoparticles can disassemble at the kidney glomerular basement membrane. Proc Natl Acad Sci U S A. 2012 Feb 21;109(8):3137–3142. 22315430 10.1073/pnas.1200718109 PMC3286910

[37] Pomin VH, Mulloy B. Glycosaminoglycans and Proteoglycans. Pharmaceuticals (Basel). 2018 Feb 27;11(1):27. 10.3390/ph11010027 PMC5874723 29495527

[38] Zhou J, Barz M, Schmid F. Complex formation between polyelectrolytes and oppositely charged oligoelectrolytes. J Chem Phys. 2016 Apr 28;144(16):164902. 27131564 10.1063/1.4947255

[39] Sponer J, Banas P, Jurecka P, et al. Molecular dynamics simulations of nucleic acids. From tetranucleotides to the ribosome. J Phys Chem Lett. 2014 May 15;5(10):1771–1782. 26270382 10.1021/jz500557y

[40] Zgarbova M, Otyepka M, Sponer J, et al. Refinement of the Cornell et al. Nucleic acids force field based on reference quantum chemical calculations of glycosidic torsion profiles. J Chem Theory Comput. 2011 Sep 13;7(9):2886–2902. 21921995 10.1021/ct200162x PMC3171997

[41] Yanao T, Koon WS, Marsden JE, et al. Gyration-radius dynamics in structural transitions of atomic clusters. J Chem Phys. 2007 Mar 28;126(12):124102. 17411103 10.1063/1.2710272

[42] Heller P, Zhou J, Weber B, et al. The influence of block ionomer microstructure on polyplex properties: can simulations help to understand differences in transfection efficiency? Small. 2017 May;13:17. 10.1002/smll.201603694 28234427

[43] Hatakeyama H, Akita H, Harashima H. The polyethyleneglycol dilemma: advantage and disadvantage of PEGylation of liposomes for systemic genes and nucleic acids delivery to tumors. Biol Pharm Bull. 2013;36(6):892–899. 23727912 10.1248/bpb.b13-00059

[44] Poon GMK, Gariepy J. Cell-surface proteoglycans as molecular portals for cationic peptide and polymer entry into cells. Biochem Soc Trans. 2007 Aug;35:788–793. 17635149 10.1042/BST0350788

