# Supplementary material for: Block catiomer with flexible cationic segment enhances complexation with siRNA and the delivery performance in vitro
Source: Sci Technol Adv Mater. 2021 Oct 13;22(1):850–63. doi: 10.1080/14686996.2021.1976055 (PMC8519541; doi:10.1080/14686996.2021.1976055)
Supplement: Supplemental Material [file TSTA_A_1976055_SM4418.pdf]

Supplementary Information

Block Catiomer with Flexible Cationic Segment Enhances Complexation with siRNA and the Delivery Performance in Vitro

Wenqian Yang[1,6], Takuya Miyazaki[1,2,3], Pengwen Chen[1,6], Taehun Hong[1,6], Mitsuru Naito[4], Yuji Miyahara[2], Akira Matsumoto[2,3], Kazunori Kataoka[6], Kanjiro Miyata[5,6] and Horacio Cabral[1,6*]

[1]*Department of Bioengineering, Graduate School of Engineering, The University of Tokyo, 7-3-1 Hongo, Bunkyo-ku, Tokyo 113-8656, Japan.*

[2]*Kanagawa Institute of Industrial Science and Technology, 705-1 Shimoimaizumi, Ebina, Kanagawa 243-0435, Japan.*

[3]*Institute of Biomaterials and Bioengineering, Tokyo Medical and Dental University, 2-3-10 Kanda-Surugadai, Chiyoda-ku, Tokyo 101-0062, Japan*

[4]*Center for Disease Biology and Integrative Medicine, Graduate School of Medicine, The University of Tokyo, 7-3-1 Hongo, Bunkyo-ku, Tokyo 113-0033, Japan*

[5]*Department of Materials Engineering, Graduate School of Engineering, The University of Tokyo, 7-3-1 Hongo, Bunkyo-ku, Tokyo 113-8656, Japan.*

[6]*Innovation Center of NanoMedicine, Kawasaki Institute of Industrial Promotion, 3-25-14, Tonomachi, Kawasaki-ku, Kawasaki 210-0821, Japan.*

Corresponding author:
Horacio Cabral
E-mail address: horacio@bmw.t.u-tokyo.ac.jp
Department of Bioengineering, Graduate School of Engineering, The University of Tokyo, 7-3-1 Hongo, Bunkyo-ku, Tokyo 113-8656, Japan

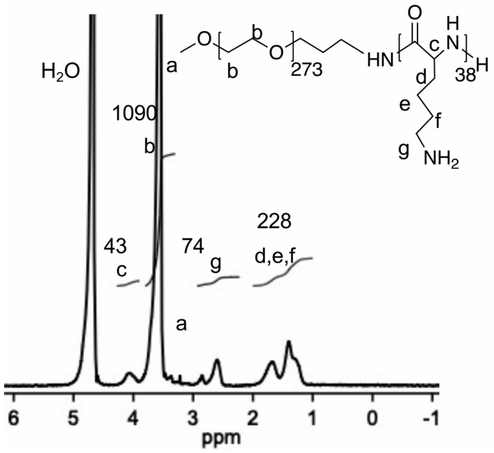

Supplementary Figure 1. ^{1}H-NMR of PEG-PLL (polymer concentration: 10 mg/mL, solvent: D_2O, and temperature: 25 °C).

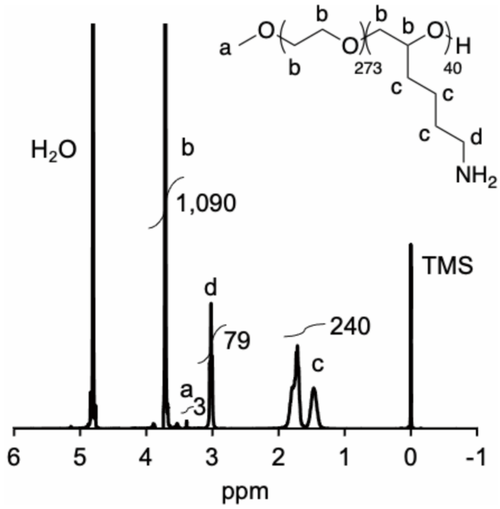

Supplementary Figure 2. ^{1}H-NMR of PEG-PGBA (polymer concentration: 10 mg/mL, solvent: D_2O, and temperature: 25 °C).

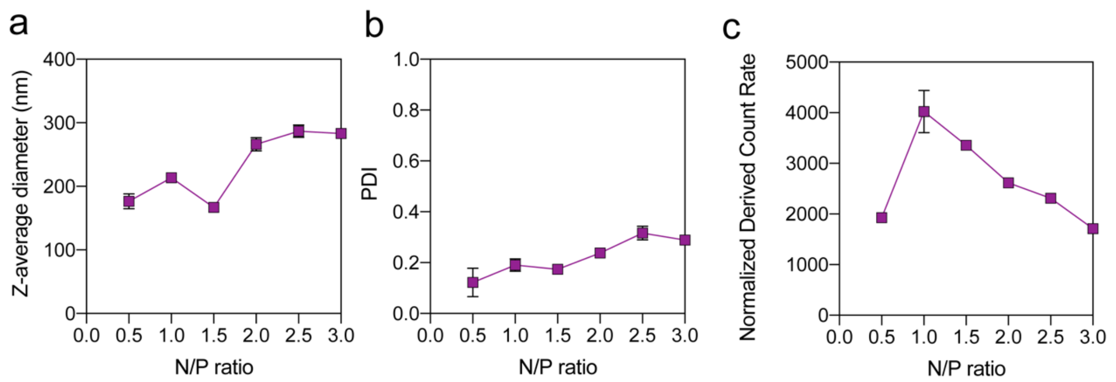

Supplementary Figure 3. DLS characterization of PIC structures from PEG-PGBA. (a) Z-average diameter, (b) polydispersity index (PDI), and (c) normalized derived count rate of PICs prepared with PEG-PGBA in 10mM HEPES and 150mM NaCl. The results are expressed as the mean ± s.d. ($n = 3$).

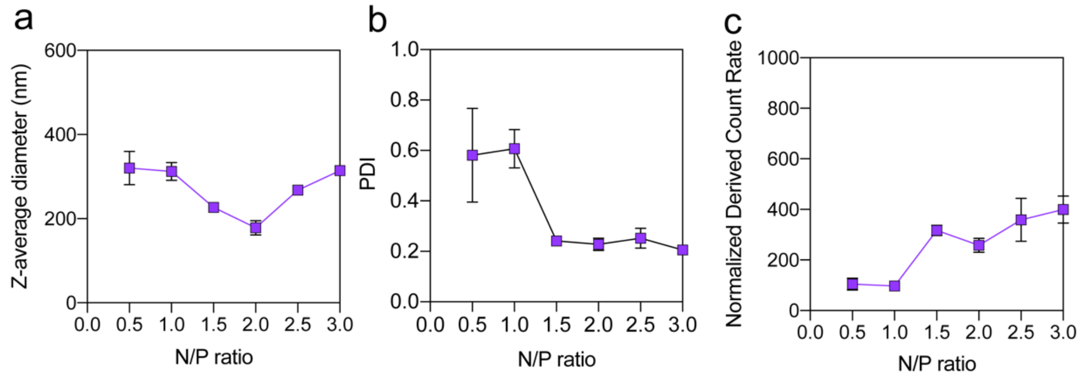

Supplementary Figure 4. DLS characterization of PIC structures from PEG-PGBA. (a) Z-average diameter, (b) polydispersity index (PDI), and (c) normalized derived count rate of PICs prepared with PEG-PGBA and diluted into a final siRNA concentration of 20 nM. The results are expressed as the mean ± s.d. ($n = 3$).

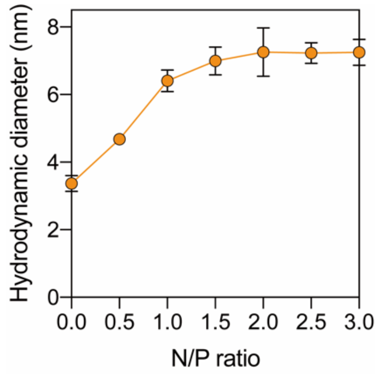

Supplementary Figure 5. The hydrodynamic diameter of PICs prepared with PEG-PLL at N/P ratio of 0.5, 1.0, 1.5, 2.0, 2.5 and 3.0. The results are expressed as the mean ± s.d. (n = 10).

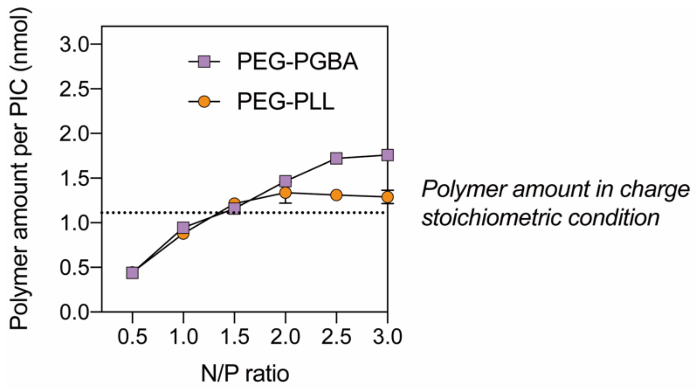

Supplementary Figure 6. The amount of polymer in siRNA PICs. The results are expressed as the mean ± s.d. (n = 3).

Supplementary Table 1. The association polymer number in uPIC (n = 10).

Sample	N/P	Average Count per molecule of polymer (mean ± s.d.)	Average Count per molecule of uPIC (mean ± s.d.)	Average association polymer number (mean ± s.d.)
PEG-PLL/siRNA	1	5.57 ± 0.17	5.79 ± 0.18	1.08 ± 0.04
PEG-PGBA/siRNA	1	5.29 ± 0.29	5.54 ± 0.92	1.05 ± 0.17

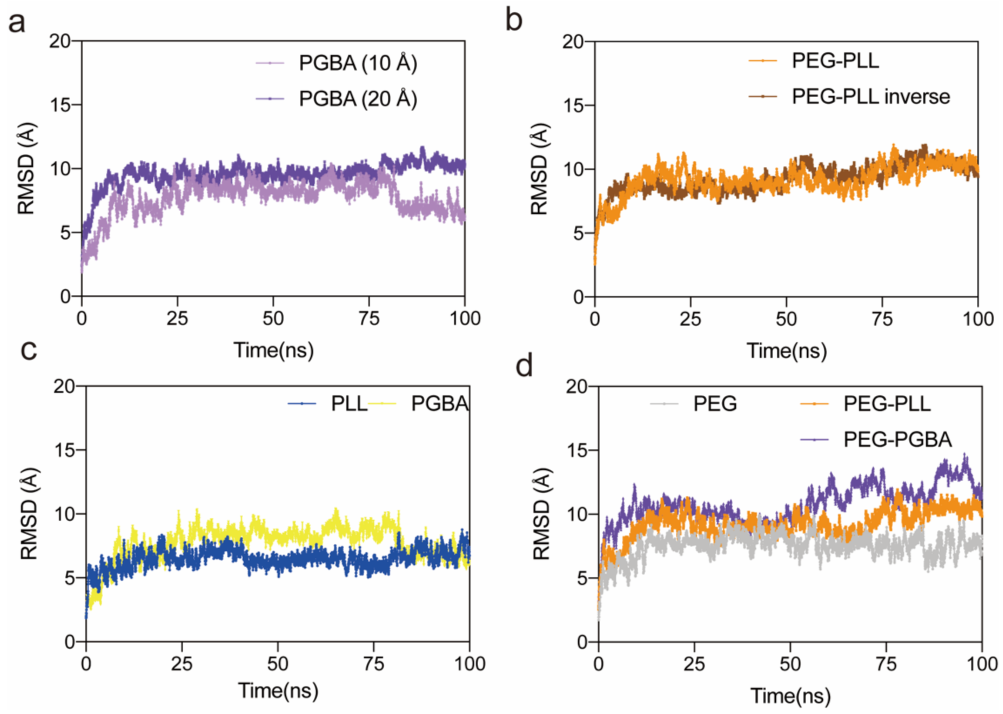

Supplementary Figure 7. Time evolution of root mean squared deviation (RMSD)of polymer/siRNA complex during molecular simulation. (**a**)Time evolution of RMSD of PGBA/siRNA at 10 Å and 20 Å. (**b**) Time evolution of RMSD of PEG-PLL/siRNA at two opposite direction. (**c**) Time evolution of RMSD of PEG-PLL/siRNA and PEG-

PGBA/siRNA. (**d**) Time evolution of RMSD of PEG, PEG-PLL and PEG-PGBA.

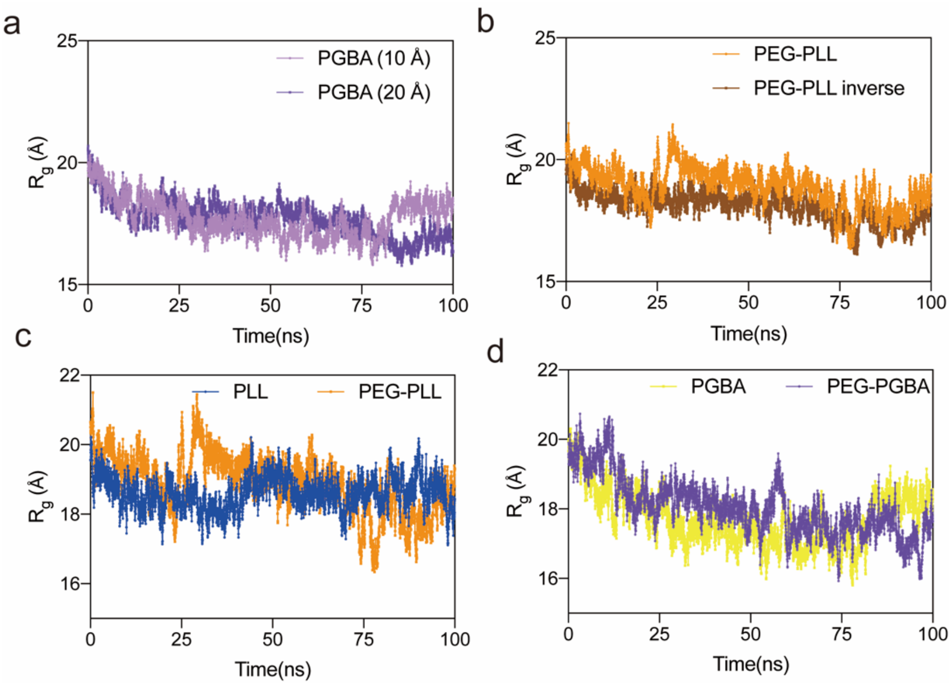

Supplementary Figure 8. Time evolution of Radius of gyration (R_g) of polymer/siRNA complex during molecular simulation. (**a**) Time evolution of R_g of PGBA/siRNA at 10 Å and 20 Å. (**b**) Time evolution of R_g of PEG-PLL/siRNA at two opposite direction. (**c**) Time evolution of R_g of PLL/siRNA and PEG-PLL/siRNA. (**d**) Time evolution of R_g of PGBA and PEG-PGBA.

Supplementary Table 2. Energy binding of PGBA/siRNA in different distance and PEG-PLL siRNA opposite direction. Energies are normalized per charged amine, expressed in kcal mol^{-1}.

Complex	ΔE_{vdw}	ΔE_{ele}	ΔE_{GB}	ΔE_{SUFR}	ΔH_{bind}
PGBA/siRNA (10 Å)	-3.800	-710.1	706.1	-0.902	-8.696
PGBA/siRNA (20 Å)	-3.125	-734.7	728.9	-0.919	-9.827
PEG-PLL/siRNA	-2.758	-638.2	636.1	-0.677	-5.612
PEG-PLL/siRNA inverse	-2.841	-718.6	714.8	-0.761	-7.384

Supplementary Table 3. Energy binding for each system in Figure 6. Energies are normalized per charged amine, expressed in kcal mol^{-1}.

Complex	ΔE_{vdw}	ΔE_{ele}	ΔE_{GB}	ΔE_{SUFR}	ΔH_{bind}
PLL/siRNA	-2.317	-725.3	720.6	-0.804	-7.875
PGBA/siRNA	-3.800	-710.1	706.1	-0.902	-8.696
PEG/siRNA	-3.224	-1.308	3.049	-0.346	-1.828
PEG-PLL/siRNA	-2.758	-638.2	636.1	-0.677	-5.612
PEG-PGBA/siRNA	-4.118	-702.4	699.3	-0.925	-8.126

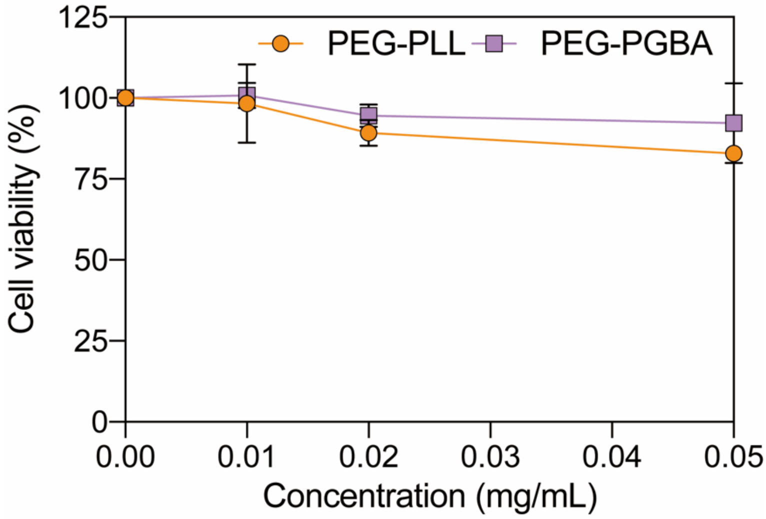

Supplementary Figure 9. Cytotoxicity of polymers studied by CCK-8 assay. Hela-luc cells were incubated with polymers for 24 h.
